# Theory of a frequency-dependent beam splitter in the form of coupled waveguides

**DOI:** 10.1038/s41598-021-84588-w

**Published:** 2021-03-03

**Authors:** Dmitry N. Makarov

**Affiliations:** Northern (Arctic) Federal University, Arkhangelsk, 163002 Russia

**Keywords:** Quantum optics, Single photons and quantum effects

## Abstract

It is known that the beam splitter in the form of coupled waveguides (BS) is one of the main devices used in quantum optics and quantum technologies. A BS has two independent parameters: one is the reflection coefficient *R* or the transmission coefficient *T*, where $R+T=1$; the second is the phase shift $\phi$. In various applications of quantum optics, these coefficients are considered constant. This is due to the fact that the frequency dependence of these coefficients is usually not taken into account, or this dependence is such that it cannot affect the constancy of these coefficients. It is shown that the coefficients *R*, *T* and phase shift $\phi$ are generally values that depend on the frequencies of incoming photons, the interaction time of photons in the BS, and the type of BS. It is established that in general, *R*, *T* and $\phi$ cannot be considered constant coefficients, and the criteria for when they can be considered constant are defined. The results obtained must be taken into account when analyzing and planning experiments where the beam splitter is presented in the form of coupled waveguides.

## Introduction

A beam splitter (BS) is one of the main devices used in quantum optics and quantum technologies. It is an essential part of many optical experimental and measurement systems, including interferometers, for example those of Michelson-Morley, Mach-Zehnder and Hong-Ou-Mandel^1–3^. In particular, the BS is used in linear optical quantum computing (LOQC)^4–6^: for example, Knill et al. in 2001 showed that it is possible to create a universal quantum computer exclusively using BSs, phase shifters, photodetectors and single photon sources (the KLM Protocol)^7^. Also, using a BS, one can create quantum entanglement between the input modes of electromagnetic fields^3,8,9^, simulate quantum transport^10^ and determine the degree of photon identity^3,11^ and others. BS is an integral part of quantum metrology^12^ and quantum information^13^, including two and multiphoton interference^3,14^. Beam splitters can be of different types. One of the most common types is a prismatic beam splitter. This type of beam splitter has a big disadvantage and is its size. It is well known that coupled waveguides can be analogous to a prismatic beam splitter. Coupling between waveguides, to realize BS-like operation, can be achieved when two waveguides are brought sufficiently close together that the evanescent fields overlap; this is known as a directional coupler (eg^15,16^). The basis of the theory of coupled waveguides (or coupled-mode theory) appeared a relatively long ago^17^, but such waveguides have been used as a beam splitter in experiments and quantum technologies relatively recently. Such a beam splitter has a significant usability advantage as it is much smaller than a prismatic beam splitter and has many other advantages^6,8,10^.

**Figure 1:**
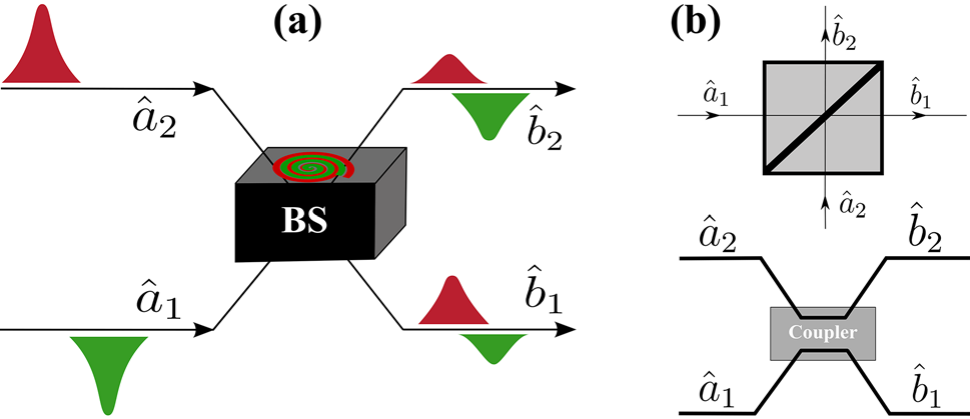
In **a** shows a beam splitter (BS) circuit with two input ports and two output ports. BS is presented as a “black box” in which the “mixing” of the input modes of the electromagnetic field takes place. In (**b**) shows BS with free-space optics, i.e. prism (top) and fiber optics, i.e. coupled waveguides (bottom).

It is well known^2,18–20^ that a lossless two-mode BS (with two input and output ports, see Fig. 1) in quantum optics is described by a unitary matrix $U_{BS}$, which has the form

1 $$\begin{aligned} U_{BS}=\begin{pmatrix} \sqrt{T}&{} e^{i\phi }\sqrt{R}\\ - e^{-i\phi }\sqrt{R}&{} \sqrt{T} \end{pmatrix} ; ~~~ \begin{pmatrix} {\hat{b}}_1\\ {\hat{b}}_2 \end{pmatrix}= U_{BS} \begin{pmatrix} {\hat{a}}_1\\ {\hat{a}}_2 \end{pmatrix}, \end{aligned}$$

where the annihilation operators 1 and 2 modes respectively represent ${\hat{a}}_1$ and ${\hat{a}}_2$, and after exiting BS ${\hat{b}}_1$ and ${\hat{b}}_2$; *T* and *R* are the coefficients of transmission and reflection; respectively, and $\phi$ is the phase shift. In the matrix $U_{BS}$, the coefficients $R+T=1$, which are often represented as $\sqrt{T}=\cos \theta$, $\sqrt{R}=\sin \theta$. Mathematically, the matrix $U_{BS}$ has two independent parameters $\theta$ and $\phi$, which are rotation angles about two orthogonal axes in the Poincare sphere. It is assumed that the physical BS can be described by any choice of $\theta$ and $\phi$, provided the correct phase shifts are applied to the outgoing modes^4,19^. It should be added that these properties are applicable only for a lossless BS. More precisely, if the losses are so small that they can be neglected. If we take into account the losses in the BS, then $R + T <1$, see^21^. Despite this, a lossless BS is one of the most important and useful devices in quantum optics. When using the $U_{BS}$ matrix in quantum optics, one is usually not concerned with the nature of the parameters *R*, *T* and $\phi$. In other words, how the statistical properties of photons in a BS in the form of coupled waveguides physically change is not important for the tasks set, i.e. BS is a black box analogue (see Fig. 1).

For a prismatic BS, the nature of the appearance of the frequency dependence of the coefficients *R*, *T* and $\phi$ is well known from classical electrodynamics. Therefore, the choice of *R*, *T* and $\phi$ in the form of constant coefficients for a prismatic BS is quite justified. For a BS in the form of coupled waveguides, these coefficients were recently found^22^. The dependence *R*, *T* and $\phi$ for a BS in the form of coupled waveguides differs from a prismatic BS and has the form

2 $$\begin{aligned} R=\frac{\sin ^2\left( \Omega t_{BS}/2 \sqrt{1+\varepsilon ^2} \right) }{(1+\varepsilon ^2)};~T=1-R;~\cos \phi =-\varepsilon \sqrt{\frac{R}{T}} ;~\varepsilon =\frac{\omega _2 -\omega _1}{\Omega }, \end{aligned}$$

where $\Omega$ is a certain frequency characterizing the BS; $t_{BS}$ is the time of interaction of photons in the BS (in the case of monochromatic and identical photons, coincides with^15^, where $R = \sin ^2(C z)$, $\phi = \pi /2$, $C = \Omega /(2v)$ is the coupling constant between adjacent waveguides, $z= v t_{BS}$, *v* is wave velocity in a waveguide); $\omega _1$ and $\omega _2$ are the photon frequencies in the first and second ports, respectively. It should be added that the greater the coupling in the waveguides, the greater the value of $\Omega$ and vice versa. Thus, we can regulate the coupling in the waveguide by changing $\Omega$. As has been shown recently in^22^, the peculiarities of the frequency dependence of *R*, *T* and $\phi$ for a BS in the form of coupled waveguides can lead to a noticeable correction of the well-known Hong-Ou-Mandel (HOM) effect. Moreover, as shown in^22,23^, this effect can be misinterpreted if the frequent dependence of *R*, *T* and $\phi$ is ignored. This is due to the fact that, in contrast to a prismatic BS, in Eq. (2) there is a resonant part when $\omega _2- \omega _1 \approx \Omega$. In this case, the coefficients *R*, *T* and $\phi$ become very sensitive to the frequency of $\Omega$. For a prismatic BS, the theory of the HOM effect remains the same^23^. This means that many studies in quantum optics, where the BS is presented in the form of coupled waveguides, must be revised taking into account the frequency dependence of *R*, *T* and $\phi$. In this paper, the general theory of a frequency dependent BS will be presented and it will be shown where it is necessary to take into account the developed theory.

## Coefficients *R*, *T* and $\phi$ in quantum optics

In general, the matrix $U_{BS}$ is needed to find the wave function of photons in the final state $\Psi _{out}$. As is well known (e.g.^14^), $\Psi _{out}=(s_1!s_2!)^{-1/2} {{{{\hat{b}}}_1}^{\dagger }}{}^{s_1} {{{{\hat{b}}}_2}^{\dagger }}{}^{s_2}|0\rangle$, where $s_1$ and $s_2$ are the initial number of photons in modes 1 and 2, respectively, $|0\rangle$ is the vacuum state, and ${{{\hat{b}}}_1}$ and ${{{\hat{b}}}_2}$ are determined from the BS matrix (1). In reality, photons are not monochromatic and the frequency distribution must be taken into account^1,2^; and in this case the initial wave function of the photons will be in the form $| \Psi _{in} \rangle = (s_1!s_2!)^{-1/2}\int \phi (\omega _1, \omega _2){{{{\hat{a}}}}^{\dagger }_1}{}^{s_1} {{{{\hat{a}}}}^{\dagger }_2}{}^{s_2} | 0 \rangle d \omega _1 d \omega _2$, where $\phi (\omega _1,\omega _2)$ is the joint spectral amplitude (JSA) of the two-modes wavefunction ($\int | \phi (\omega _1, \omega _2) |^2 d \omega _1 d \omega _2 = 1$). Taking into account the distribution of frequencies, $\Psi _{out}=(s_1!s_2!)^{-1/2} \int \phi (\omega _1, \omega _2){{{{\hat{b}}}_1}^{\dagger }}{}^{s_1} {{{{\hat{b}}}_2}^{\dagger }}{}^{s_2}|0\rangle d\omega _1 d \omega _2$; its well-known expression for $\Psi _{out}$ will have a broader meaning given the resulting *R*, *T* and $\phi$, which depend on the frequencies $\omega _1,\omega _2$. In other words, in general, when modeling various circuits in quantum optics, the coefficients *R*, *T* and the phase shift $\phi$ cannot be considered constant.

In order to find $\Psi _{out}$ and determine clear criteria when *R*, *T* and $\phi$ in quantum optics can be constant values, and when it is necessary to take into account the frequency dependence of these coefficients, consider in more detail the value of $\Omega$ to Eq. (2). It was shown in the works^22,23^ that $\Omega$ in the general case $\Omega = 8 \pi n \mathbf{u}_1 \mathbf{u}_2/(\omega _1 + \omega _2)$, where *n* average concentration of electrons interacting with electromagnetic fields in two BS ports, $\mathbf{u}_1$ and $\mathbf{u}_2$ polarization of photons in 1 and 2 ports, respectively. Also in^22,23^ this frequency was estimated and shown that it is one of the most important characteristics in a BS in the form of coupled waveguides.

Next, we define the bounds when *R*, *T* and $\phi$ can be considered constant coefficients in $\Psi _{out}$. For simplicity, let us consider a specific type of $\phi (\omega _1, \omega _2)$ in $\Psi _{out}$ that is suitable for most of the photon sources used^24^:

3 $$\begin{aligned} \phi (\omega _1,\omega _2)\propto e^{-\frac{(\omega _1+\omega _2-\Omega _p)^2}{2\sigma ^2_p}}e^{-\frac{(\omega _1-\omega _{01})^2}{2\sigma ^2_1}}e^{-\frac{(\omega _2-\omega _{02})^2}{2\sigma ^2_2}}. \end{aligned}$$

Equation (3) allows us to analyze the value of $\Psi _{out}$ for two cases that are of practical interest. The first case is spontaneous parametric down-conversion (SPDC): for example, for $\Omega _p = \omega _{01}+\omega _{02}; \sigma _1 = \sigma _2 = \sigma$ is SPDC of type I, where $\sigma _p$ is the bandwidth of the pump beam, $\sigma$ the bandwidth for both the signal and the idle beams. If we consider $\sigma _p \rightarrow \infty$ in (3), then this will be the case of Fock states (e.g.^24^). Indeed, in this case, in Eq. (3), the $\phi (\omega _1,\omega _2)$ function will be factorized, which corresponds to Fock states. It is easy to show that when we integrate over frequencies for $\omega _{02}-\omega _{01}\ll \omega _{01}, \omega _{02}$; $\omega _{01}/\sigma _1\gg 1$; $\omega _{02}/\sigma _2\gg 1$ and $\Omega \gg \sigma _{1}, \sigma _{2}$ we get $\varepsilon =\Delta /\Omega$, where

4 $$\begin{aligned} \Delta =\omega _{02}\frac{\sigma ^2_p+2\sigma ^2_1}{\sigma ^2_1+\sigma ^2_2+\sigma ^2_p}-\omega _{01}\frac{\sigma ^2_p+2\sigma ^2_2}{\sigma ^2_1+\sigma ^2_2+\sigma ^2_p}+\Omega _p \frac{\sigma ^2_2-\sigma ^2_1}{\sigma ^2_1+\sigma ^2_2+\sigma ^2_p},  \\ \Omega =\frac{4\pi n}{\omega _0 } \mathbf{u}_1 \mathbf{u}_2;~~\omega _0=\frac{2\sigma _p\sigma ^2_1\sigma ^2_2+\sigma ^2_p(\sigma ^2_2\omega _{01}+\sigma ^2_1\omega _{02})}{4\sigma ^2_1\sigma ^2_2+(\sigma ^2_1+\sigma ^2_2)\sigma ^2_p},~~~~~~ \end{aligned}$$

where in the case of SPDC of type I and Fock states $\Delta =\omega _{02}-\omega _{01}$. As a result, we obtain the coefficients *R*, *T* and the phase shift $\phi$, in (2) as constant values (i.e. *R*, *T* and $\phi$ will retain their forms in Eq. (2), where $\Omega$ and $\varepsilon$ are constant). It should be added that the conditions $\omega _{02}-\omega _{01}\ll \omega _{01}, \omega _{02}$; $\omega _{01}/\sigma _1\gg 1$; $\omega _{02}/\sigma _2\gg 1$, under which *R*, *T* and $\phi$ have become constant, are quite natural for most photon sources used in optical quantum computing. It should be added that the condition Eq. (4) essentially represents the condition for the monochromaticity of photons, that is. when the frequency “spread” can be ignored and it tends to zero.

Consider when the condition $\Omega \gg \sigma _ {1}, \sigma _{2}$ is satisfied. The frequency $\omega _0$ under the condition $\omega _{02}-\omega _{01}\ll \omega _{01}, \omega _{02}$; $\omega _{01}/\sigma _1\gg 1$; $\omega _{02}/\sigma _2\gg 1$ will be of the order of $\omega _0 \sim \omega _{01}, \omega _{02}$, then $\Omega \sim \frac{4 \pi n}{\omega } \mathbf{u}_1 \mathbf{u} _2$ ($\omega \approx \omega _{01}, \omega _{02}$). As shown in^22^, the value of *n* can be any value depending on how tightly the waveguides are coupled. Let’s choose the maximum possible value of $n_{max}$ for evaluation. In this case, $n_{max}$ will be equal to the average concentration of electrons in the waveguides. In this case, it can be shown that $\Omega _{max} \sim \omega ^2_p / \omega$ ($\omega _p$ is the plasma frequency). For example, for solids and optical photons, it is easy to obtain that the condition $\Omega _{max} \gg \sigma _{1}, \sigma _ {2}$ will be satisfied. This means that the *R*, *T* and $\phi$ coefficients will always be constant in quantum optics and their frequency dependence (for optical photons) can be ignored when the waveguides are strongly coupled to each other. If we consider the case of higher frequencies, then the frequency dependence for *R*, *T* and $\phi$ must be taken into account. In the case of a sufficiently weak coupling in the waveguides, the frequency dependence of the *R*, *T* and $\phi$ coefficients must be taken into account. There are no strict boundaries when to use the fixed *R*, *T* and $\phi$ coefficients, and when to take into account the frequency dependence. However, you can always evaluate $\Omega$ and compare them with $\sigma _{1}, \sigma _{2}$ to make a conclusion about the frequency dependence of *R*, *T* and $\phi$.

**Figure 2:**
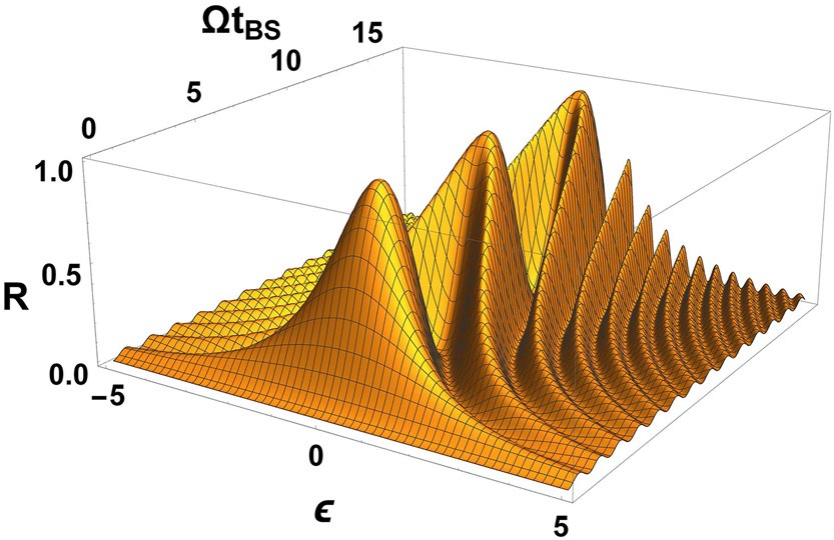
The reflection coefficient *R* is presented depending on two parameters included in it: $\varepsilon$ and $\Omega t_{BS}$. A constant value of *R* can only be selected if there is a certain dependency $\varepsilon =\varepsilon (\Omega t_{BS})$ (horizontal section in the figure). Figure are made in Wolfram Mathematica 12 software.

**Figure 3:**
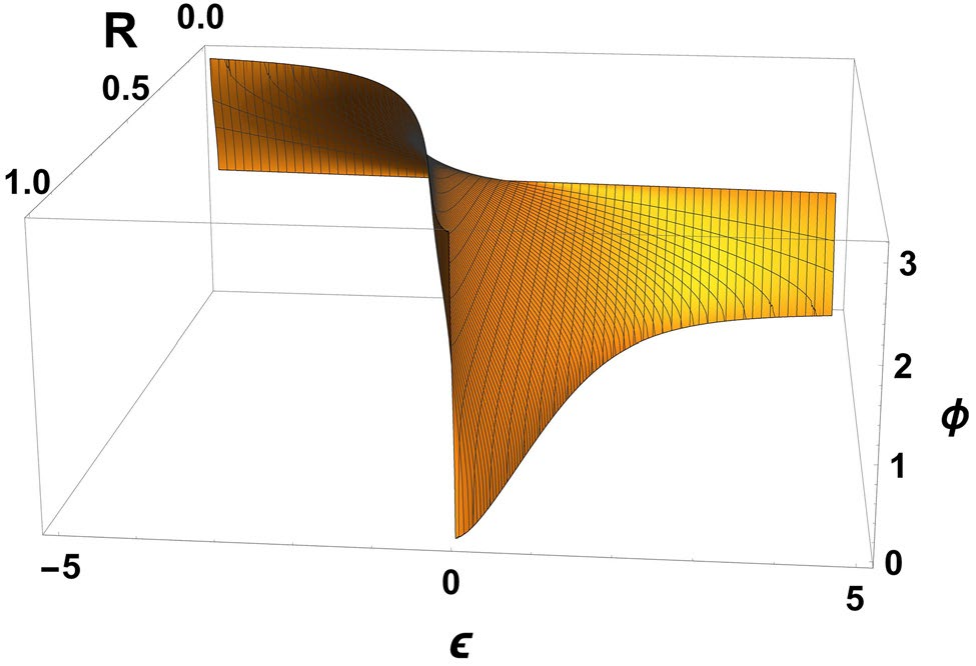
The phase shift $\phi$ is represented as being dependent on two parameters *R* and $\varepsilon$. By fixing the value *R* and making a horizontal slice at the specified $\phi$, we obtain a point that defines the value $\varepsilon$ required for these parameters. Figure are made in Wolfram Mathematica 12 software.

Often, in quantum optics, $R, T=1-R$ and $\phi$ are independently selected by constant coefficients depending on the tasks posed. We show that choosing $\phi$ in an arbitrary way for constants *R*, *T* needs to be done very carefully. The coefficients *R*, *T* can be set to constant values when $\omega _{02}-\omega _{01}\ll \omega _{01}, \omega _{02}$; $\omega _{01}/\sigma _1\gg 1$; $\omega _{02}/\sigma _2\gg 1$ and $\Omega \gg \sigma _{1}, \sigma _{2}$ for this you need to choose two parameters as constant values, these being $\Omega t_{BS}$ and $\varepsilon$. The phase shift is defined from (2) as $\cos \phi =-\varepsilon \sqrt{\frac{R}{T}}$ and has a single value for the given $\Omega t_{BS}$ and $\varepsilon$. If in any quantum-optical circuits it is necessary to set the BS to the constant values of *R*, *T* and $\phi$ selected for the scheme, this can be done by varying the parameters $\Omega t_{BS}$ and $\varepsilon$ (see Fig. 2), and the necessary phase shift $\phi$ can be selected by changing $\varepsilon$ (see Fig. 3). This means that the phase shift at constant *R*, *T* can be selected by changing the characteristics of the photons used $\omega _{01}, \omega _{02}$ or the type of BS i.e. $\Omega$.

Here we have considered the case when *R*, *T* and $\phi$ can be considered constant. In general, it is possible to obtain the necessary $\Psi _{out}$ in quantum optical without setting *R*, *T* and $\phi$ as constants. This means that in each BS in the quantum optical scheme, you can change $t_{BS}$, and $\Omega$ with the selected photon source, or change the photon source as well, simulating the necessary $\Psi _{out}$ at the output of the scheme. It is quite simple to model, since the matrix BS $U_{BS}$ and the coefficients *R*, *T* and the phase shift $\phi$ are of a simple analytical form (2).

Wave function for monochromatic photons $\Psi _{out}=(s_1!s_2!)^{-1/2} {{{{\hat{b}}}_1}^{\dagger }}{}^{s_1} {{{{\hat{b}}}_2}^{\dagger }}{}^{s_2}|0\rangle$, as shown in^25^, can be found analytically $\Psi _{out}=\sum ^{s_1+s_2}_{k=0}c_{k,s_1+s_2-k}|k,s_1+s_2-k\rangle$, or, up to an insignificant phase factor for calculating the probability $\Psi _{out}=\sum ^{s_1+s_2}_{k=0}\sqrt{\lambda _k (R)}|k,s_1+s_2-k\rangle$ where $\lambda _k (R)=\left| c_{k,s_1+s_2-k}\right| ^2$,

5 $$\begin{aligned} &c_{k,p}=\sum ^{s_1+s_2}_{n=0}A^{s_1,s_2}_{n,s_1+s_2-n}A^{*{k,p}}_{n,s_1+s_2-n}e^{-2in {\arccos \left( \sqrt{1-R} \sin \phi \right) }} , \\ &A^{k,p}_{n,m}=\frac{\mu ^{k+n}\sqrt{m!n!}}{(1+\mu ^2)^{\frac{n+m}{2}}\sqrt{k!p!}}P^{(-(1+m+n), m-k)}_{n}\left( -\frac{2+\mu ^2}{\mu ^2} \right) ,  \\&\mu =\sqrt{1+\frac{1-R}{R}\cos ^2\phi }-\cos \phi \sqrt{\frac{1-R}{R}}, \end{aligned}$$

where $P^{\alpha , \beta }_{\gamma }(x)$ are Jacobi polynomials, $s_1$ and $s_2$ are the number of photons in the first and second input ports, respectively, *k* and *p* are the number of photons in the first and second output ports, respectively. Moreover, the condition $k + p = s_1 + s_2$ is satisfied, i.e. the number of photons in the system does not change^25^, $|k,s_1+s_2-k\rangle =|k\rangle |p\rangle$ is the state of the photons at the output ports of the BS. It should be added that the $\lambda _k (R,\phi )$ parameter is the Schmidt mode and is the probability of detecting the system in the $|k,s_1+s_2-k\rangle$ state^25–27^. As shown in^25^, the Schmidt parameter $\lambda _k(R)$ does not depend on the phase shift $\phi$ regardless of its choice in Eq. (5). This is a very convenient property for calculating various physical characteristics in a BS.

In the case of non-monochromatic photons, as mentioned above, we obtain

6 $$\begin{aligned} \Psi _{out}=\sum ^{s_1+s_2}_{k=0}\int \phi (\omega _1, \omega _2)\sqrt{\lambda _k (R)}| k, s_1+s_2-k \rangle d\omega _1 d \omega _2. \end{aligned}$$

In this case, the probability ${{\overline{\lambda }}}_k$ to detect *k* and $s_1 + s_2-k$ on the first and second ports, respectively, will be determined

7 $$\begin{aligned} {{\overline{\lambda }}}_k = \int |\phi (\omega _1, \omega _2)|^2\lambda _k (R) d\omega _1 d \omega _2. \end{aligned}$$

**Figure 4:**
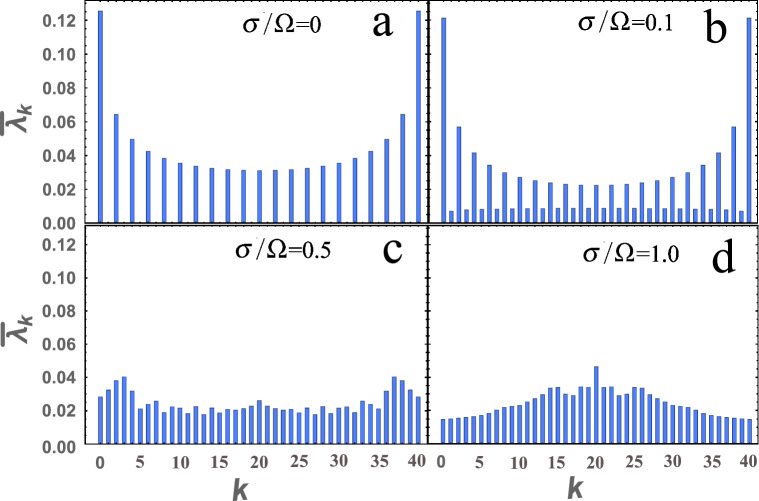
A histogram of the dependence of the probability ${{\overline{\lambda }}}_k$ of detecting *k* and $p = s_1 + s_2-k$ photons at the output of the first and second ports, respectively, is presented. The selected states, where $s_1 = 20$ and $s_2 = 20$ for ${\overline{R}} = {\overline{T}} = 1/2$ and for four values $\sigma / \Omega = (0; 0.1; 0.5; 1.0)$. Figure are made in Wolfram Mathematica 12 software.

Next, we will show how the probability of detecting *k* and $p = s_1 + s_2-k$ photons will look like, respectively, at the first and second ports of the BS, taking into account the frequency-dependent BS. To do this, consider an example where the photons are identical ($\omega _{01}=\omega _{02}=\omega$ and $\sigma _1=\sigma _2=\sigma$) and $\sigma /\omega \ll 1$, but not monochromatic, i.e. when $\sigma / \Omega$ can be arbitrary. It should be added that the case where the BS was not frequency-dependent was considered in the article^19^. Let us show, as an example, how the frequency dependence of a BS can strongly change the statistics of photons, see Fig. 4. In Fig. 4 presents the photon statistics for ${\overline{R}} = {\overline{T}} = 1/2$, where ${\overline{R}} = \int R (\omega _1, \omega _2) | \phi (\omega _1, \omega _2) |^2 d \omega _1 d \omega _2$. In the case when the photons are monochromatic, i.e. when $\sigma / \Omega \ll 1$, our result coincides with the previously known^19^, see Fig. 4a. It should be added that the result obtained here for monochromatic photons coincides with^19^ not only for this particular case, but for all.

Figure 4 shows that the $\sigma / \Omega$ parameter greatly changes the statistics of photons at the output ports of the beam splitter. If in the Fig. 4a we observe only even photons, then on others (b, c, d) there are also odd ones. Moreover, for $\sigma / \Omega \sim 1$ in the photon statistics, there is absolutely no similarity with the statics for small $\sigma / \Omega \ll 1$. This is quite an important conclusion, since such states, an example of which is given here $| s, s \rangle$, i.e. with the same number of photons at the input of the BS and with the reflection coefficient $R = 1/2$, are the Holland-Burnett (HB) states^28^. It is well known that this states is of great interest in various fields of physics, for example, in quantum metrology^12,29^.

One of the striking examples where a developed theory can substantially correct known results is the calculation of the correlation function $\Gamma _{1,2}$. It is well known that $\Gamma _{1,2}$ is an important characteristic in quantum optics. This value is calculated and measured experimentally in the HOM effect^3^, in various quantum-optical schemes and measurements^30^. In the case of monochromatic photons at ports 1 and 2 of the BS, this is a function, as is well known by $\Gamma _{1,2} =|U_{1,1}U_{2,2}+U_{1,2}U_{2,1}|^2=|R-T|^2$, where $U_{i,j}$ are elements of the matrix BS (1). If we consider non-monochromatic photons, i.e. using the spectral amplitude $\phi (\omega _1, \omega _2)$ (JSA), then

8 $$\begin{aligned} \Gamma _{1,2} =\int \left( |\phi (\omega _1,\omega _2)|^2(R^2+T^2)-2\mathrm{Re} \left\{ T R \phi (\omega _1,\omega _2)\phi ^*(\omega _2,\omega _1)\right\} \right) d\omega _1 d\omega _2. \end{aligned}$$

Which is easy enough to get from Eq. (6) for $s_1 = 1, s_2 = 1$, see e.g.^3,22,31–33^. If we choose the parameters in $\phi (\omega _1, \omega _2)$ and $\Omega$ such that *R*, *T* can be considered constant (e.g. for $\phi (\omega _1, \omega _2)$ presented in Eq. (3), this is $\omega _{02} - \omega _{01} \ll \omega _{01}, \omega _{02}$; $\omega _{01}/ \sigma _1 \gg 1$; $\omega _{02} / \sigma _2 \gg 1$ and $\Omega \gg \sigma _{1}, \sigma _{2}$) we get $\Gamma _{1,2} = | R-T |^2 =| {\overline{R}}-{\overline{T}} |^2$. Otherwise, $\Gamma _{1,2}$ is defined Eq. (8). If we choose $R = T = 1/2$ (same as ${\overline{T}} = {\overline{R}} = 1/2$) in the general case $\Gamma _{1,2} \ne 0$. This is a rather important conclusion, because using this condition $R = T = 1/2$ it is usually assumed that $\Gamma _{1,2} = 0$ (e.g. HOM effect in the case of identical photons). In practical implementation, such a case appears when $\Omega$ is quite small (e.g. in the case of Eq. (3) when $\Omega \sim \sigma _ {1}, \sigma _ {2}$). This case can be realized, for example, for BS in the form of a coupled waveguides^22^. It is also interesting to note that in the case of monochromatic and identical photons, i.e. if in the Eq. (4) parameter $\varepsilon = 0$ value $\Gamma _ {1,2} = \cos ^2(\Omega t_{BS})$ in Eq. (8) matches $\Gamma _{1,2} = \cos ^2(2Cz)$ in^15^(in this work $R = \sin ^2(C z)$, $\phi = \pi /2$, where $C = \Omega /(2v)$ is the coupling constant between adjacent waveguides, $z= v t_{BS}$, *v* is wave velocity in a waveguide). In the case of non-monochromatic but identical photons ($\omega _{01}=\omega _{02}$ and $\sigma _1=\sigma _2=\sigma$), choosing $\phi (\omega _1,\omega _2)$ as Eq. (3) and using the conditions $\omega _{02} - \omega _{01} \ll \omega _{01}, \omega _{02}$; $\omega _{01}/ \sigma _1 \gg 1$; $\omega _{02} / \sigma _2 \gg 1$, you can get

9 $$\begin{aligned} \Gamma _{1,2} =\frac{1}{\sqrt{2\pi }}\int ^{\infty }_{-\infty } e^{-y^2/2}\left( 1-\frac{2\sin ^2\left( \Omega t_{BS}/2 ~\sqrt{1+(\frac{\sigma }{\Omega })^2 y^2}\right) }{1+(\frac{\sigma }{\Omega })^2 y^2} \right) ^2 dy . \end{aligned}$$

Can also be obtained using Eq. (9), provided $\Omega t_ {BS} \rightarrow \infty$ and $\sigma t_ {BS} \rightarrow \infty$, simple expression

10 $$\begin{aligned} \Gamma _{1,2} =1+\frac{3}{4}\left( \frac{\Omega }{\sigma }\right) ^2-\frac{\sqrt{2\pi }}{8}\left( \frac{\Omega }{\sigma }\right) ^3 e^{\left( \frac{\Omega }{\sqrt{2}\sigma }\right) ^2}\left( 3+5\left( \frac{\sigma }{\Omega }\right) ^2\right) \mathrm{erf} \left( \frac{\Omega }{\sqrt{2}\sigma }\right) , \end{aligned}$$

where $\mathrm erf(x)$ this is an error function. I should add that Eq. (10) can be obtained with $\Omega t_ {BS} \rightarrow \infty$ and $\sigma t_ {BS} \rightarrow \infty$ if we do not take into account in Eq. (9) oscillating terms (i.e. with sine and cosine). Already at $\Omega t_{BS} \sim 100$ and $\sigma t_{BS} \sim 100$, the error of this calculation is less than 1 $\%$. From Eq. (9) it can be seen that if the photons are monochromatic, i.e. $\sigma / \Omega \ll 1$ (strictly speaking, the condition $\sigma t_ {BS} \ll 1$ must also be satisfied), then we get the case described above, where $\Gamma _ {1,2} = \cos ^2(\Omega t_{BS})$. The importance of using frequency-dependent *R*, *T* and $\phi$ coefficients in quantum physics is clearly seen.

**Figure 5:**
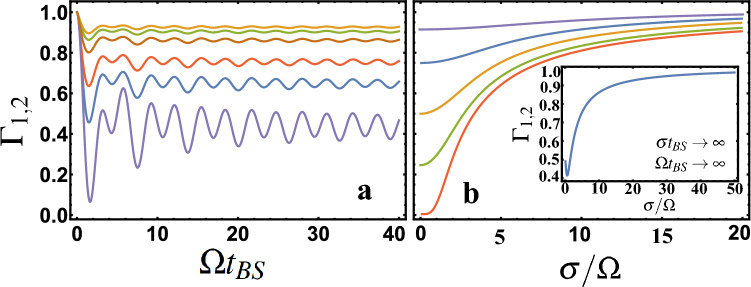
The dependence of the correlation function $\Gamma _{1,2}$ is presented. (**a**) shows the dependence on $\Omega t_{BS}$ for six values of $\sigma / \Omega = (1; 3; 5; 10; 15; 20)$ (bottom to top). (**b**) shows the dependence on $\sigma / \Omega$ for five values of $\Omega t_{BS} = (\pi / 10; \pi / 6; \pi / 4; \pi / 3; \pi / 2)$ (top-down). The inset in (**b**) illustrates Eq. (10). Figure are made in Wolfram Mathematica 12 software.

Figure 5a shows that the correlation function $\Gamma _{1,2}$ is very different from $\Gamma _{1,2} = \cos ^2(\Omega t_{BS})$, see^15^. From Fig. 5b you can also see that $\Gamma _{1,2}$ strongly depends on the $\sigma / \Omega$ parameter if we take into account that for $\Gamma _ {1,2} (\sigma / \Omega = 0)$ the theory presented here coincides with the previously known^15^. In the case when $\Gamma _{1,2} = 0$, i.e. when quantum interference (HOM effect) occurs, it can be realized only for $\sigma / \Omega \rightarrow 0$ and $\Omega t_{BS} = \pi / 2 + \pi n$ (where $n = 0,1, 2 \ldots$ are integers). If we consider $\sigma / \Omega > 0$, then $\Gamma _{1,2} \ne 0$ and this can be seen from Eq. (9). Although for $\sigma / \Omega > 0$ the correlation $\Gamma _{1,2}$ can be quite small and quantum interference is significant, this can be seen, for example, from Fig. 5a, where $\sigma / \Omega = 1$ and $\Omega t_ {BS} = \pi / 2$. It should be added that the analysis of quantum correlations using the $\Gamma$ correlation function is well known, not only for bosonic statistics, but also for fermionic^34,35^.

## Discussion and conclusion

The developed theory is an essential addition to the BS theory on coupled waveguides, since the frequency dependence of the reflection coefficient *R* and the phase shift $\phi$ is taken into account. We have shown that the results obtained are not only of theoretical interest, but they also have practical applications in quantum optics. It should be added that frequency-dependent BS is one example where frequency can be important. For example, waveguide lattices^36,37^ can also be frequency dependent and such studies are interesting in the future. Moreover, using aligned waveguides, i.e. considered here BSs, you can implement quantum gates^15,16^, which can also be frequency-dependent.

The results obtained have well-known limiting cases. For example, when we consider monochromatic photons, our theory for calculating $\Psi _{out}$ coincides with^19^, and for calculating the correlation function, taking into account the identity of photons, it coincides with^15^. If the length $z = v t_{BS}$ of the waveguide is too small (more precisely, the value of $\Omega t_{BS}\ll 1$ is too small) or $\Omega \rightarrow 0$ (or $\Omega \ll \omega _2- \omega _1$), then the reflection coefficient is $R \rightarrow 0$. This means that photons move along the waveguides independently of each other, without being reflected.

It should be noted that the resulting expressions in Eq. (2) have their own specifics with respect to classical expressions. The main role here is the identity of photons (when $\omega _2- \omega _1 \ll \omega _1, \omega _2$). Even in the case of a very weak interaction i.e. when $\Omega$ is small, the electromagnetic field modes can exchange energy at $(\omega _2- \omega _1) / \Omega \lesssim 1$, where as a result *R* and $\phi$ are not small. In other words, the energy exchange is resonant in nature, i.e. when the frequencies are close, so that $(\omega _2- \omega _1) / \Omega \lesssim 1$, the energy exchange takes place intensively. This is what causes the very “mixing” of photons depicted in Fig. 1. That is why, in quantum optics for BS based on coupled waveguides, it is necessary to take into account the frequency dependence of the reflection coefficients *R*, transmission *T* and phase shift $\phi$.
